# 

**Published:** 1896-10-31

**Authors:** 


**Notices of Births, Deaths, and Marriages** are inserted in this column at a charge of 2s. 6d. for an announcement not exceeding four lines.

### NOTICE TO CORRESPONDENTS.

All MS., Letters, Books for Review, and other matters intended for the Editor should be addressed The Editor, "The Hospital," The Hospital Building, 28 & 29, Southampton Street, Strand, W.C.

The Editor cannot undertake to return rejected MS., even when accompanied by stamped directed envelope.

### Notice to Advertisers and Subscribers.

All Advertisements, Orders for Copies of The Hospital, and Business Communications should be addressed to **THE MANAGER, (Not to The Eitdor),** The Hospital, The Hospital Building, 28 & 29, Southampton Street, Strand, London, W.C.

The Cost of Subscription, post free, is as follows:—Per week, 2½d.; per quarter, 2s. 8d.; per half-year, 5s. 3d.; per annum, 10s. 6d. Foreign :—Per annum, 15s. 2d.; payable, in all cases, in advance.

### IMPORTANT NOTICE.

The EDITORIAL and PUBLISHING OFFICES of "THE HOSPITAL" are now TRANSFERRED to their new premises, THE HOSPITAL BUILDING, 28 & 29, SOUTHAMPTON STREET, STRAND, LONDON, W.C.

**NOTICE.—Vol. XX. of "The Hospital" (April to September, 1896), in handsome cloth binding, is now on sale at the Office of this Journal, price 7s. 6d. Cases for binding the Numbers contained in this Volume 1s. 6d. Index to Vol. XX., 2½d., post free. The Monthly Part for November will be ready on November 2nd, Price 6d., by post 8d.**

### Books Received.

James Maclehose and Sons.
"Manual of Diseases of the Ear." By Thomas Barr, M.D.

Swan Sonnenschein and Co.
"The Diary of a Resurrectionist." By James Blake Bailey, B.A.

Whittaker and Co.
"The Art of Cooking for Invalids." By Florence B. Jack.

**Periodicals and Pamphlets.**—The New Saturday, Sunday at Home, Girl's Own Paper, Boy's Own Paper, Leisure Hour, Sunday Hours, Lady's Realm, Clinical Journal, Tri-State Medical Journal, Medical Week, Review of Reviews, London, Charity Organisation Review, American Journal of the Medical Sciences, Medical Mission Quarterly, Guy's Hospital Gazette, Columbus Medical Journal, Literary Digest, Chicago Clinical Review, Medical Press, Vectis, Therapist, British Review, Scalpel, Health Magazine.

### THE STUDENT OF MEDICINE.

### APPOINTMENTS.

R. D. Boase, L.R.C.P.Lond., M.R.C.S.Eng., appointed Medical Officer of Health to the Penzance Town Council. K. C. Chetwood-Aiken, M.B., C.M., appointed Junior House Surgeon to the Royal London Ophthalmic Hospital. Dr. Durrant, appointed Medical Officer of Health to the Oxenden Rural District Council. Dr. W. F. Fisher, appointed Medical Officer for the Ramsey District of the Huntingdon Union. T. D. Forbes, M.B., appointed Assistant Medical Officer to the Bethnal Green Union. H. E. Girdlestone, M.R.C.S., L.R.C.P., appointed House Surgeon to the Royal Eye Hospital, Southwark. L. Glover, M.D.Cantab., appointed Medical Officer to the Out-patients at the Hampstead Hospital. G. W. Gostling, M.B.Lond., appointed House Surgeon to the County Hospital, York. H. Head, M.A , M.D.Camb., L.R.C.P , appointed Assistant Physician to the London Hospital. G. J. Johnston, M.A., M.B., B.Ch., Assistant Surgeon to the Richmond Hospital, appointed Surgeon to the City of Dublin Hospital. H. Jones, M.D., appointed Assistant Surgeon to the Main Bridewell, Liverpool. D. Knocker, M.R.C.S.Eng., L.R.C.P.Lond., appointed Resident Medical Officer to the Tower Hamlets Dispensary. J. M. MacCormac, M.D.Durh , L.R.C.P.&S.Edin., appointed Physician to the Victoria Hospital for Diseases of the Nervous System, Belfast. C. D. Murray, M.B., C.M.Edin., appointed Physician to the Victoria General Hospital, Halifax, Nova Scotia. G. S. Page, L.R.C.P., L.R.C.S.Edin., appointed Medical Officer to the St. Peter's Hospital of the Bristol Incorporation. E. Parsons, L.D.S.R.C.S.Eng., appointed Dental Surgeon to the Metropolitan Hospital, Kingsland, N.E. R. F. Symons, M.R.C.S., L.R.C.P., appointed Assistant Medical Officer to the Metropolitan Asylums Board Fountain Hospital, Grove Road, Lower Tooting. E. G. Walls, M.R.C.S., L.R.C.P.Lond., appointed Medical Officer for the Revesby District of the Horncastle Union. E. E. Ware, M.B.,Lond., L.R.C.P.Lond., M.R.C.S.Eng., appointed Medical Officer to the Hampstead Hospital. C. Woakes, M.R.C.S., L.R.C.P., appointed Assistant Surgeon to the London Throat Hospital.

### PASS LIST.

### Conjoint Board in England.

The following gentlemen passed the second examination of the Board in the subjects indicated on Monday, October 12th :—

Anatomy and Physiology.—N. A. Aylmers-Hughes, Yorkshire College, Leeds; H. F. Sheldon and J. Miller, Owens College, Manchester; S. Smith, University College, London, and St. Mungo's College, Glasgow; L. S. Smith, Mason College, Birmingham; P. Southan, Mason College, Birmingham, and Mr. Cooke's School of Anatomy and Physiology; H. Hemsted and P. G. Stock, University College, Bristol; E. J. Scorah, Firth College, Sheffield; M. Sheehan, Queen's College, Cork; and R. Lamb, University College, Liverpool.

Anatomy only.—F. C. Torbett, Owens College, Manchester; R. Holt, Royal College of Surgeons of Ireland and Mr. Cooke's School of Anatomy and Physiology; O. C. Sibley, Middlesex Hospital and Mr. Cooke's School of Anatomy and Physiology.

Physiology only.—L. C. Martin, Guy's Hospital and Mr. Cooke's School of Anatomy and Physiology; E. Hyde, Cambridge University and St. George's Hospital; P. G. Williams, St. Thomas's Hospital; T. L. Braidwood, St. Thomas's Hospital and Mr. Cooke's School of Anatomy and Physiology.

Eighteen gentlemen were referred in both subjects, four in anatomy only and three in physiology only.

Tuesday, October 13th.

Anatomy and Physiology.—W. F. Bennett, C. C. B. Thompson, C. C. C. K. White, W. C. Douglass, and H. Bond, St. Bartholomew's Hospital; B. S. Jones and L. S. Dudgeon, St. Thomas's Hospital; F. W. Twort, St. Thomas's Hospital and Mr. Cooke's School of Anatomy and Physiology; R. N. Watson, Westminster Hospital; E. H. B. Stanley and A. J. Sheldon, University College, London; F. C. Rogers and R. E. B. Wilmot, St. Mary's Hospital; and H. B. Dismorr, Guy's Hospital.

Twenty-two gentlemen were referred on both subjects.

Wednesday, October 14th.

Anatomy and Physiology.—H. Goodman, W. G. Hamilton, S. Mason, H. G. Pinker, A. B. Pugh, A. J. W. Wells, and H. G. Wood-Hill, St. Bartholomew's Hospital; R. Winterbotham, F. S. Leech, F. W. James, E. B. L. Moore, and H. C. Woodyatt, University College, London; W. B. Harris, W. C. C. C. Davies, W. L. Baker, and W. Holmes, St. Mary's Hospital; J. A. MacLeod, London Hospital; H. B. Foster and W. McIlroy, Guy's Hospital.

Twenty-three gentlemen were referred in both subjects.

Thursday, October 13th.

Anatomy and Physiology.—W. F. Peach, J. F. E. Bridger, and P. N. Smith, St. Mary's Hospital; R. W. H. Meredith and A. B. Vines, Middlesex Hospital; E. G. Battiscombe, London Hospital; O. E. Lemin, London Hospital and Mr. Cooke's School of Anatomy and Physiology; F. E. Manning and A. P. Watkins, University College, London; and T. W. Smith, Charing Cross Hospital.

Anatomy only.—H. A. Ahrens and A. H. Safford, King's College, London; J. H. Tripe, London Hospital and Mr. Cooke's School of Anatomy and Physiology; and T. McCarthy, Queen's College, Cork, and Mr. Cooke's School of Anatomy and Physiology.

Physiology only.—H. M. Leaths, St. Thomas's Hospital; R. Pistoujee, Ceylon Medical College; H. N. Clarke, Cambridge University and London Hospital; and V. S. A. Bell, Cambridge University and St. Bartholomew's Hospital.

Eighteen gentlemen were referred in both subjects, three in anatomy only and one in physiology only.

